# One-week multidisciplinary post-graduate palliative care training: an outcome-based program evaluation

**DOI:** 10.1186/s12909-020-02200-7

**Published:** 2020-08-18

**Authors:** Piret Paal, Cornelia Brandstötter, Johannes Bükki, Frank Elsner, Anna Ersteniuk, Elisabeth Jentschke, Andreas Stähli, Iryna Slugotska

**Affiliations:** 1 WHO Collaborating Centre, Institute for Nursing Science and Practice, Paracelsus Medical University, Salzburg, Austria; 2 Palliative Care Center at the Cantonal Hospital, St. Gallen, Switzerland; 3 Department of Palliative Medicine, RWTH Aachen University, Aachen, Germany; 4 Ivano-Frankivsk National Medical University, Ivano-Frankivsk, Ukraine; 5 Psycho-oncological, palliative-psychological and gerontological department, Comprehensive Cancer Center of the University Hospital, Wuerzburg, Germany; 6 Johannes-Hospiz Muenster, Munster, Germany; 7 Regional Clinical Palliative Care Centre, Ivano-Frankivsk, Ukraine; 8 Family Medicine Department, Ivano-Frankivsk National Medical University, Ivano-Frankivsk, Ukraine

**Keywords:** Education, Training, Palliative care, Multidisciplinary, Post-graduate, Curriculum, Self-assessment, Response bias, Ukraine

## Abstract

**Background:**

A multi-professional, post-graduate, one-week palliative care training program was piloted in November 2019 at the University of Ivano-Frankivsk, Ukraine. A formal evaluation of this program was performed.

**Methods:**

This is a comparative, retrospective outcome-based evaluation of an educational intervention. Participants completed evaluation forms at the end of the course (post-intervention = T1), covering demographics, comparative retrospective self-assessment (40 items, 6-point Likert scale), organizational aspects, and general feedback (free text). At T1, the responses represent actual self-assessment, pre-interventional (T0) scores were generated by retrospective self-assessment. The Retrospective Performance Gain (RPG) was calculated on group level for the comparative self-assessment, demographic and organizational variables were analyzed by descriptive statistics, and free text answers were processed by qualitative methodology (content analysis).

**Results:**

Fifty-three of 56 attendants from all professions relevant to palliative care completed the evaluation forms (response 94,6%), with mean age 39y (22–64) and mean working experience 13,6y (1–44). Overall ratings of the program were very positive. Comparative retrospective self-assessment demonstrated a marked RPG from T0 to T1 on all items. Free text comments emphasized the need for regular nation-wide educational programs and for further education in bereavement care; inter-professional practice; communication; palliative care philosophy; professional self-care; specific nursing skills; dementia care; and advocacy, while the general contribution of the program to palliative care development in Ukraine was acknowledged.

**Conclusions:**

Systematic evaluation of a post-graduate international training program in palliative care may provide a mutual learning experience and map country-specific barriers and facilitators that have to be addressed when setting up palliative care services.

## Background

The World Health Organization (WHO) has expressed high interest in globally promoting the development and implementation of palliative care services [1–3]. The Astana Declaration on Primary Health Care (2018) states that “promotive, preventive, curative, rehabilitative services and palliative care must be accessible to all.” [4] In the WHO European region the developments in palliative care have been monitored closely. The most frequently used indicators to monitor palliative care developments at country level are: 1. the number of palliative care services per population; 2. the existence of a palliative care national plan, strategy or program; 3. an established medical specialty “palliative care”; 4. the availability and allocation of funds for palliative care; 5. medical schools teaching palliative care in undergraduate curricula; and 6. the total use of opioids-morphine equivalents [5]. The Atlas of Palliative Care in Europe (2019) has indicated that while most countries have established some kind of legal framework for the provision of palliative care, only in twelve out of 53 countries patients’ palliative care needs are routinely screened by primary care, meaning that most countries provide palliative care only in the last month of life [6]. It has been the long-standing credo of the palliative care community to facilitate and offer basic palliative care training to all medical and nursing students. This suggestion is not followed uniquely by national governments, politicians and other key stakeholders [7]. Despite all efforts, implementing a palliative care training program is rarely part of a national strategy, but rather depends on largely individual non-governmental advocacy [8].

To enhance and enable palliative care education, networking and setting global standards is of particular significance [6, 9–12]. Previous international training initiatives, such as the European Certificate in Essential Palliative Care (ECEPC) [13], the Transformational Palliative Nursing Leadership Program for CEE countries [14], and several other cross-country educational collaborations have proven very successful [11]. However, before setting up programs, it is essential to acquire “the knowledge and skills” as well as “the cultural sensitivity and the humility to provide long-term, effective, locally relevant palliative care training and technical assistance.” [11] In 2018 an ethnographic fieldwork to Central Asian, Eastern and South-Eastern European countries was conducted. Palliative care leaders and educators from 23 countries unanimously agreed that the main barriers to the development of educational work are limited political interest, insufficient educational structures, missing curricula, lack of trainers, and structural constraints of the health care system. They equally agreed that a European recommendation for a multidisciplinary post-graduate training would be of great importance [15].

In Ukraine, palliative and hospice care for adults and children started to evolve with the help from NGOs, supported by international grants from donors affiliated with Soros’s Open Society Foundations [16]. According to Tymoshevska and Shapoval-Deinega (2018), the first hospices in Ukraine were established in 1996–1997 in Lviv, Korosten, and Ivano-Frankivsk, the latter being the oldest operating hospice in Ukraine. The first palliative care association was established in 2006 to raise awareness about the need for palliative care [17]. Per annum about six thousand people suffering from cancer, HIV/AIDS, tuberculosis, diabetes and other life-limiting conditions, are estimated to be in need of palliative care. Despite a growing number of hospice beds, only 15% of seriously ill people die in healthcare facilities due to limited access to specialized medical care and proper pain management [18]. Palliative care needs among elderly people dying of old age and struggling with frailty or neuro-cognitive disorders, such as dementia, have not been assessed so far. The majority of patients requiring palliative care are dying at home without adequate social and psychological support or symptom relief. However, an explicit home-based care plan has not been established yet, which means that “patients and their carers remain alone with their problems such as chronic pain syndromes, organ disorders, lack of care and psychological support, loneliness, social isolation and insecurity due to inadequate coordination of the work of the healthcare institutions, an insufficient amount of hospices and multidisciplinary teams.” [18]

Describing the formation and development of palliative care in Ukraine, Shunkina and Hromovyk (2018) point out the insufficient number and inappropriate professional level of specialists in palliative care. Additionally, they acknowledge that the level of undergraduate and postgraduate palliative care is unsatisfactory. Specialists working in palliative care institutions may acquire relevant and necessary knowledge mainly through trainings, seminars, conferences held by the public and charitable organizations. This clearly emphasizes the need for palliative care training as well as international collaboration in the field of education. To support the development of palliative care in Ukraine, a one-week multidisciplinary post-graduate on-site training program was designed following the results of a survey conducted among European palliative care educators and clinicians [10]. The program was piloted at the Ivano-Frankivsk National Medical University (IFNMU) in Ukraine in November 2019 at the kind request of local collaborators. This study was designed to evaluate the effects of a one-week multidisciplinary post-graduate palliative care training and to map the learning curve using a comparative outcome-based self-assessment strategy [19] administered at the end of the training week.

## Methods

### Study design

For this study the comparative retrospective self-assessment strategy was chosen. The method of data collection relied on asking course participants to evaluate the course and assess their palliative care competencies based on a novel self-assessment strategy at the end of the training week.

### Setting

The overall aim of this course was to promote the idea of timely integration of palliative care services, encourage networking and communication across the disciplines, and enhance self-care, self-reflection, and team building. The course was organized by the WHO Collaborating Centre at the Paracelsus Medical Private University (PMU) in Salzburg, Austria, the Johannes-Hospice Academy in Muenster, the IFNMU and the local hospice in Ivano-Frankivsk, Ukraine. Teaching modules were chaired by international faculty (Johannes-Hospice Academy in Muenster, Interdisciplinary Palliative Care Centre at the University Hospital of Wuerzburg, both in Germany; Centre of Palliative Care in St. Gallen, Switzerland).

The course was free of charge. Since this is a preliminary project serving as a pilot with the intent to be modified based on teachers’ experiences and participants’ feedback, no formal certification by professional associations for Continuous Medical Education (CME) was obtained [10, 11, 15].

This study design was approved by the IFNMU Bioethics Commission (10 October 2019, No.110/19).

### Sample

Participants (*n* = 56) were recruited from hospitals, city clinics, hospices, palliative units and mobile home care teams including physicians, oncologist, psychologists, nurses, social workers and chaplains directly working with patients. The attendants represented different regions of Ukraine, namely: Ivano-Frankivsk, Kyiv, Lviv, Cherkasy, Kharkiv, Zakarpattia.

### Intervention

A one-week, multi-disciplinary, multi-professional post-graduate palliative care program titled “Capacity Building and Empowerment” was hosted in November 2019 in Ivano-Frankivsk, Ukraine. This one-week course was initiated to support palliative care education in West-Ukraine. Ukrainian associates, the IFNMU and the local hospice arranged the venue, invited the participants, and provided hospitality services to a multi-professional faculty (including medical, nursing, and psychological backgrounds plus a program coordinator). Overall, the event consisted of lectures, group work, self-reflection exercises, and topic-related discussions; furthermore, there were profession-specific workshops and an exercise of multidisciplinary decision making based on two case studies proposed by Ukrainian colleagues. Classes were Monday - Friday from 9 a.m. to 5 p.m. The course entailed 40 h of classroom teaching, which were divided into following course modules:

- Principles of Palliative Care (8 modules)
- Management of complex symptoms (16 modules)
- Diagnosis and handling of demented patients, relatives of palliative patients, and those in grief and mourning (6 modules)
- Self-care, team-building factors, communication (10 modules)

All course materials, such as specific learning goals and supporting literature, were integrated into a curriculum template and made available online (whocc.pmu.ac.at/toolkit). PowerPoint slides, classroom activities, and questions for self-assessment were designed to match the goals of each module. All course-related materials, including the course evaluation form, were translated into Ukrainian prior to the course and published online. The entire program was held in German with consecutive Ukrainian translation. A certificate of attendance was issued by the PMU. Participants were encouraged to use and adopt all provided materials in their work related to palliative care, where appropriate. Participants’ feedback and comments helped to contextualize and adapt the content to the Ukrainian setting, which made the one-week training a mutual learning and teaching experience.

### Data collection and measures

All participants were informed about the course evaluation procedure. At the end of the course, they were asked to complete paper-based evaluation forms. Besides demographics (7 items), organizational aspects (7 items), and two open questions (free text answers), the core element of the survey was the comparative retrospective self-assessment ((40 items, 6-point Likert scale from 1 (fully agree) to 6 (completely disagree), Table 1). Essentially, comparative retrospective self-assessment implies

- a self-assessment of attendants’ abilities after the intervention (at the end of the course, = T1) AND
- a retrospective self-assessment at baseline (beginning of the course, = T0) as perceived at T1.

**Table 1:** The Retrospective Performance Gain of knowledge, skills and attitudes

	Item	T0 Mean (retrospective)	T1 Mean	Retrospective Performance Gain (%)
(1)	I can explain the meaning of a life-limiting illness to the person concerned	3.09	1.93	55.83%
(2)	I can explain the Total Pain concept in detail	3	2	50%
(3)	I can name essential characteristics of fear, demoralization and depression	3.19	1.98	55.25%
(4)	I can explain the main social needs of those affected in the context of their impact on palliative care	3.38	2.25	47.48%
(5)	I can explain the meaning of rituals at the end of life	3.42	2.08	55.37%
(6)	I can name risk factors of family caregivers	3.28	1.96	57.89%
(7)	I can explain the relevant legal and ethical principles of care for seriously ill and dying patients	3.32	2.06	54.31%
(8)	I can name all outpatient and inpatient palliative care structures	3.40	2.17	51.25%
(9)	I can substantiate the importance of interdisciplinary and inter-professional cooperation	3.09	1.89	57.42%
(10)	I can name all the relevant risk factors of burnout development process	2.92	1.68	64.58%
(11)	I adopt the palliative care approach as early as possible in the disease process	3.4	2.08	55%
(12)	I develop individual strategies to actively support the patient’s wellbeing and quality of life to maintain the patient’s dignity	3.17	1.94	56.68%
(13)	I recognise health care supply related resources and risks related to individual family structures	3.06	1.83	59.71%
(14)	I follow explicitly the fundamentals of various consulting and communication methods	3.17	2	53.92%
(15)	I identify possible questions of meaning and conscience related to imminent death	3.38	2.17	50.84%
(16)	I involve members and associates in discussions and decision-making processes	3.09	2.13	45.93%
(17)	I make and implement decisions in the patient’s and relatives’ interests	2.43	1.72	49.65%
(18)	I coordinate necessary support and care options both within and beyond the team	2.94	2.12	42.27%
(19)	I definitely use context- and person appropriate vocabulary	2.30	1.62	52.31%
(20)	I routinely implement self-care strategies	2.63	1.85	47.85%
(21)	I always consider the patient and their family members as experts in their own lives	2.74	1.92	47.13%
(22)	I always perceive and acknowledge the patient’s individual symptom perception and suffering experience	2.51	1.64	57.62%
(23)	I can evaluate patient’s psychological symptoms in a structured way	3.11	2.17	44.55%
(24)	I can identify my personal challenges and limitations in the presence of patients and their relatives	3.32	2.49	35.78%
(25)	I always support the bereavement and loss processes of those affected	4.02	2.81	40.07%
(26)	I provide professional security and confirmation to caregivers and relatives	3.04	2.17	42.65%
(27)	I always allow family members and relatives to be involved in the decision-making process	2.81	2.19	34.25%
(28)	In the palliative care setting, I generally promote multiprofessional teamwork	2.91	1.94	50.79%
(29)	In my professional role, I always respond adequately to the emotional reactions of interviewees	3.02	2.11	45.05%
(30)	I routinely build up an appreciative error culture	3.06	2.08	47.57%
(31)	I always use my own resources cautiously and team-oriented	2.98	2.13	42.93%
(32)	I essentially respect my own and others’ limits	2.53	1.92	39.87%
(33)	I implement a high degree of self-reflection in all areas of my professional activity	3.23	2.25	43.95%
(34)	I differentiate professionally my own values from those of the patient	2.51	1.72	52.32%
(35)	I regularly reflect on my own meaning of life	2.11	1.62	44.14%
(36)	I respect professionally the existing social system (family and friends)	2.09	1.66	39.45%
(37)	I always respect the autonomous decisions of the patient and their relatives and friends	2.23	1.75	39.02%
(38)	I always show the willingness to develop and seek conflict solutions	2.30	1.64	50.77%
(39)	I always give praise and criticism within and beyond the team with tact	2.65	1.85	48.48%
(40)	I apprehend that up-to-date knowledge is not static and needs to be continuously developed and expanded in the process of lifelong learning	1.98	1.54	44.90%

The 40-item novel self-assessment strategy was translated from German into Ukrainian and integrated into the evaluation form. The English items presented in this paper are directly translated from German.

This comparative self-assessment strategy has been developed by the Educational Working Group of the German Society for Palliative Medicine (DGP) and used with their written approval. According to our knowledge, this is the first time this particular instrument was used to assess participants’ learning outcomes in the post-graduate setting.

Initially, the comparative outcome-based evaluation strategy was developed to assess undergraduate medical students’ competencies [19]. Compared to more established pre-post self-assessment strategies, participants in comparative self-assessment are asked to rate their initial performance level retrospectively [20]. This strategy has been described as “participant friendly” and estimated to be sufficient to generate an adequate appraisal of learning outcome. Furthermore, higher response rates may be expected if self-ratings of initial and final performance levels are collected at one single time-point [20]. In their study, the investigators found out that the “collection of true pre-test ratings is not required” (pre-test ratings include one self-assessment at the beginning and one at the end of a learning session) as there is no significant difference found between the two methods [20].

Such a comparative retrospective self-assessment strategy has been tested and approved also in a post-graduate palliative care setting. A study to evaluate an intensive palliative care faculty development program at Harvard Medical School demonstrated that using retrospective rather than conventional pre- versus post-program ratings to measure change in degree of preparation did not change the number of statistically significant findings [21].

The comparative retrospective self-assessment was administered on the final day of the one-week training, e.g. at the end of the course. Participants were kindly asked to assess their performance level at the beginning of the course retrospectively (T0) as well as the actual performance level after (T1) the training session [20]. All items of the self-assessment highlighted the topics discussed during the one-week multi-professional palliative care training. Participation was voluntary and anonymous. By completing and returning the evaluation forms, the participants gave their written consent to participate in this study.

### Measures

For the quantitative analysis, all data were entered into SPSS (IBM Statistics, Version 25). For this study the calculation was conducted on the group level. Therefore, the mean score for T0 and T1 per item was calculated.

The “Retrospective Performance Gain [%]” formula was adapted for data analysis [20]:

$$ \mathrm{Retrospective}\ \mathrm{Performance}\ \mathrm{Gain}\ \left[\%\right]=\frac{\mu_{\mathrm{thentest}}\hbox{-} {\mu}_{\mathrm{posttest}}}{\mu_{\mathrm{thentest}}\hbox{-} 1}\times 100 $$

This formula is the modification of the comparative self-assessment (CSA)-Gain formula presented earlier [19]:

$$ \mathrm{CSA}\ \mathrm{gain}\ \left(\%\right)=\frac{\mu_{\mathrm{pre}}-{\mu}_{\mathrm{post}}}{\mu_{\mathrm{pre}}-1}\times 100 $$

This study defined the gain in knowledge, skills and attitudes that occurred during a module “as the difference in mean ratings (pre/post) within a student cohort enrolled in the module.” [19] This means that “μ thentest” stands for the mean value of the retrospective self-assessment of knowledge, skills and attitudes before the course and “μ posttest” stands for the mean value of the self-assessment after the course.

The evaluation of organizational aspects and the questions regarding the overall course framework were analyzed using descriptive statistics.

The free-text sections of the course evaluation were analyzed applying qualitative content analysis [22] by two independent researchers experienced with qualitative data analysis. The participant feedback and appraisal are presented by frequency and visualized as graphical summary.

## Results

### Demographical data

Of the 56 attendants, 53 completed and returned the evaluation forms (response rate, 94.6%). Forty-four (83%) of the respondents were female and nine (17%) male. They were between 22 and 64 years old (mean 39 years). The majority of them (*n* = 32) were married or living with a partner (*n* = 3). Ten persons were living alone (18.9%), four were divorced (7.5%) and four widowed (7.5%). The majority of participants worked full time (*n* = 47, 88.7%), only six (11.3%) reported working part-time. The professional backgrounds were as follows: physicians (*n* = 24, 45.3%), nurses (*n* = 11, 20.8%), psychologists (*n* = 7, 13.2%), chaplains (*n* = 4, 7.5%), physiotherapists (n = 3, 5.7%), social workers (n = 2, 3.8%), and other (n = 2, 3.8%). Fifty participants (3 missing) reported on their working experience, which was between one and 44 years (median 13,6 years). Denominations were as follows: Ukrainian Orthodox (*n* = 36, 67.9%), Catholic (n = 4, 7.5%), Protestant (n = 1, 1.9%), and other (*n* = 8, 15.1%). Four participants reported not belonging to a religious community (7.5%).

### Comparative retrospective self-assessment

In Table 1, all items, the mean scores of T0, T1, and the Retrospective Performance Gain [%] are shown. Due to the fact that 1 represents “totally agree” and 6 “totally disagree,” lower scores mean that palliative care competencies are self-rated higher. The calculation indicates the group mean values; thus the individual performance gain may differ.

### Organizational aspects

Participants stated that the course was very well (*n* = 43, 81.1%) or well (*n* = 9, 17%) organized (missing: *n* = 1). The overall framework of the training week was very good (*n* = 40, 75.5%) or good (12, 22.6%) (missing: *n* = 1). The training week was structured very good (*n* = 45; 84.9%), good (*n* = 5, 9.4%) or predominantly not good (*n* = 1, 1.9%) (missing: *n* = 2). The scope of contents of the training was mainly assessed as very useful (41, 77.4%), useful (*n* = 9, 17%), or satisfactory (*n* = 1, 1.9%) (missing: *n* = 2). The overall impression of the training week was very good (*n* = 38, 71.7%), good (*n* = 13, 24.5%), or satisfactory (*n* = 1, 1.9%) (missing: *n* = 1). The teachers were evaluated as very competent (*n* = 46, 86.8%) or competent (*n* = 5, 9.4%) (missing: *n* = 2).

### Feedback and constructive criticism

The overall feedback (47 responses) from participants was exceedingly positive. Participants acknowledged the openness and high professionalism of the faculty and were confident that the course will be a great contribution to the development of palliative care in Ukraine. All participants expressed the importance of multidisciplinary training in palliative care. The most common single request (*n* = 15) was to offer such training opportunities across the entire country. Participants indicated the need to learn and understand the philosophy of palliative care in order to convey this important message to local community leaders and politicians (*n* = 4). A wish for collaboration in palliative care research was expressed by one participant.

**Fig. 1:**
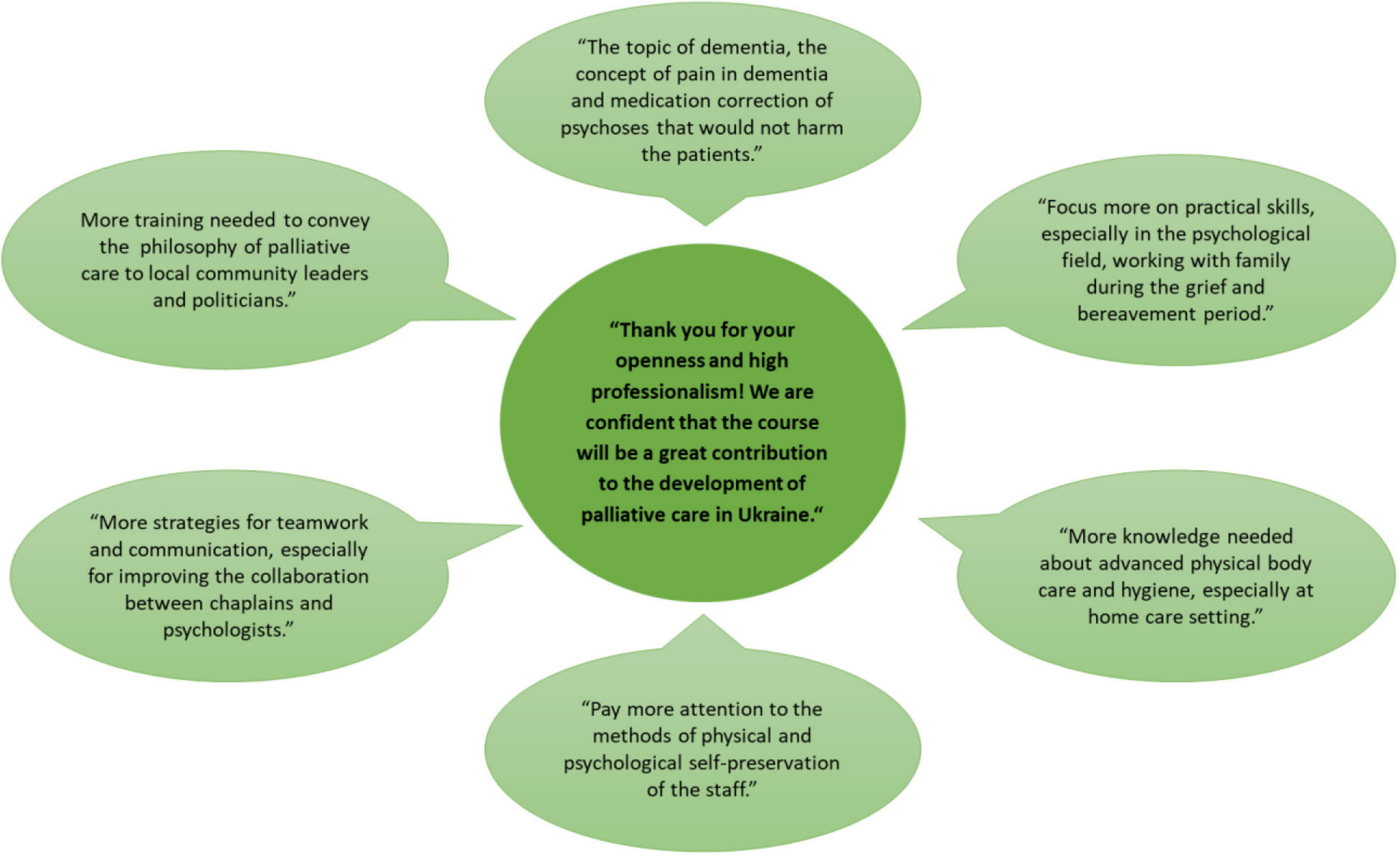
An overview of participants’ feedback and constructive criticism

In terms of content of the training program, as shown in Fig. 1, participants preferred a focused approach rather than practical skills (*n* = 8), especially psychologists who were supporting families during grief and bereavement. Equally, participants were keen on issues of teamwork (*n* = 7), communication, and improving inter-professional collaboration (e.g. between chaplains and psychologists). Participants also requested to emphasize methods of physical and psychological self-care of staff more strongly (*n* = 5). In terms of practical nursing skills, they wished to gain more knowledge about advanced physical body care and hygiene, especially in the home care setting (5 times). Moreover, the topics of dementia care, pain management in dementia, and safe pharmacological management of mental health problems were addressed (*n* = 3). Regarding organization, one participant suggested taking five minute brakes after every 40 min, while another one suggested tackling practical skills in small groups rather than in the plenary.

## Discussion

### Summary of findings

The overall aim of this brief training program was to promote the idea of timely integration of palliative care services, encourage networking and communication across the disciplines, and enhance self-care, self-reflection, and team building skills. Palliative care clinicians from all relevant backgrounds (medical, nursing, and psycho-social professionals) were included. The content and organization of this course was well-received. Even though most of the participants had lots of work experience (median 13,6y), they perceived a significant benefit in terms of skills, knowledge, and attitudes. By comparative retrospective self-assessment, a significant retrospective performance gain in all major items of the program was reported. Additional free-text comments suggest that this gain was most pronounced regarding theoretical knowledge while participants expressed an ongoing need for improving practical skills in nursing, dementia care, bereavement support, communication, and team building. Further need for nationwide training opportunities were suggested by a number of participants.

### Discussion of literature

These findings expressed by experienced palliative care clinicians reflect the educational needs regarding palliative care in Ukraine. On a national level, barriers and facilitators have been described in detail [17]. While pain and pain-related symptom control to reduce suffering is still the most established and monitored aspect of palliative care [3], current developments toward the early integration of palliative care indicate the need for complex symptom assessment and management competencies across the healthcare settings [23]. This means paying attention to social challenges, spiritual or existential suffering, fear, grief and loss of dignity, feeling of being a burden, or suffering from social isolation and loneliness. Supporting patients and families and integrating their values, beliefs, and preferences, has to be brought into the focus of palliative care advocacy, education and service provision [24, 25]. For example, in Ukraine, taking care of the elderly has long been seen as a family obligation. The healthcare and social care system needs to recognize that families and should not be left alone taking care of patients with such a high symptom burden and care need. Any chronic condition (such as dementia) is affecting caregivers as well, with adverse physical, mental, social, and economic effects [26]. Supporting patients and their families to reduce health-related suffering is a societal challenge that needs to be encouraged using global strategies [27].

### Limitations

First of all, although professional interpreters were always at hand, the language barrier may have hindered direct communication and spontaneous interaction as not all participants were able to express themselves in English or German. More important, meaning of a specific term may differ between Ukrainian and German language – for example, among the Ukrainian attendants, nurses might classify themselves as “medical” while in other languages this term is restricted to physicians.

Secondly, the current German, Ukrainian and English versions of the comparative retrospective self-assessment have not been validated formally in any of these languages. Moreover, this strategy was in fact used for the first time in the post-graduate setting. Given this setback, it proved to provide rapid yet profound insights into a novel international palliative care training program, thus, enabling faculty to promptly respond to comments and make subsequent adaptations of the curriculum.

Thirdly, when interpreting the self-assessments it is important to be aware that within this formula “the large net increase in self-assessment from 5.0 to 3.0 would produce the same gain (50%) as the much smaller net increase from 2.0 to 1.5.” [19].

Finally, the selected retrospective approach might threaten the validity of results due to response shift or effort justification bias. Highly motivated participants who have invested considerable resources in completing a course might be prone to overestimating their learning outcome, which may lead to ‘effort justification bias’ [20, 28, 29].

### Strengths

Self-assessment is an essential component of educational programs as it may increase the interest and motivation levels of students for the subjects, leading to enhanced learning and better academic performance as well as helping them develop critical skills for the analysis of their own work [30]. In post-graduate education, however, self-assessment has not played a major role despite a strong educational merit [31]. While clinicians’ skills improve during training, they often do not receive sufficient feedback concerning their performance. Thus, self-assessment may be a valuable means to improve performance and motivation [31, 32].

### Implications for clinical care

There is a number of lessons learned from both conducting the program and its evaluation. Country-specific challenges have to be taken into account and addressed actively. A successful program is dependent on the faculty’s awareness of the local situation. In Ukraine, the long prevailing medical tradition prohibits discussing the aspects of life-limiting disease with patients. Hence, discussing patients’ and families’ preferences and making plans accordingly has very little weight in current palliative care practice. Patients and families are used to physician-centered decision making, which challenges integrating a multi-professional and interdisciplinary approach. Social services traditionally are seen as protectors of children’s rights while psychotherapy is offered only to cancer patients on a regular basis. Currently, patients with life-limiting conditions and their families have very low priority and most of the services provided to this group come with high out of pocket payments. To change the situation participants indicated the urgent need for team-building skills and communication strategies. Equally important is the ability of faculty members to encourage, give advice, and support attendants in setting aspirational goals. Such goals may be seen on a societal, organizational or individual level, all equally relevant for sustaining the change. In this study, participant feedback confirmed that discussing the phenomenon of loss, grief, and bereavement within palliative care deserved special attention. While mourning was still widely associated with religious communities and specific rituals in Ukraine, participants were highly motivated to enhance work across professional borders, in particular, between psychologists and chaplains, to extend loss and grief-oriented services, and organize support groups for family caregivers.

## Conclusions

Post-graduate, international educational collaboration in palliative care is mutually beneficial. Systematic evaluation of a program may reveal specific barriers and facilitators regarding palliative care. Thus, education may offer the opportunity to gain in-depth knowledge of country-specific challenges and to share experience about possible solutions. The global aim of such programs is to establish sustainable advocacy networks, educational programs, and clinical services in palliative care on local, regional and national levels.

## Data Availability

The curriculum template and the comparative retrospective self-assessment strategy in Ukrainian and German languages are made available at whocc.pmu.ac.at/toolkit. The dataset for the current study are available from the corresponding author on reasonable request.

## Abbreviations

WHO: World Health Organization
CEE: Central and Eastern Europe
CME: Continuous Medical Education
DGP: German Society for Palliative Medicine
ECEPC: European Certificate in Essential Palliative Care
IFNMU: Ivano-Frankivsk National Medical University
PMU: Paracelsus Medical University in Salzburg, Austria
RPG: Retrospective Performance Gain
